# Toward industrial scale synthesis of ultrapure singlet nanoparticles with controllable sizes in a continuous gas-phase process

**DOI:** 10.1038/srep15788

**Published:** 2015-10-29

**Authors:** Jicheng Feng, George Biskos, Andreas Schmidt-Ott

**Affiliations:** 1 Department of Chemical Engineering, Delft University of Technology, NL-2628, BL Delft, The Netherlands; 2 Faculty of Civil Engineering and Geosciences, Delft University of Technology, NL-2628 CN, Delft, The Netherlands; 3 Energy Environment and Water Research Centre, The Cyprus Institute, Nicosia 1645, Cyprus

## Abstract

Continuous gas-phase synthesis of nanoparticles is associated with rapid agglomeration, which can be a limiting factor for numerous applications. In this report, we challenge this paradigm by providing experimental evidence to support that gas-phase methods can be used to produce ultrapure non-agglomerated “singlet” nanoparticles having tunable sizes at room temperature. By controlling the temperature in the particle growth zone to guarantee complete coalescence of colliding entities, the size of singlets in principle can be regulated from that of single atoms to any desired value. We assess our results in the context of a simple analytical model to explore the dependence of singlet size on the operating conditions. Agreement of the model with experimental measurements shows that these methods can be effectively used for producing singlets that can be processed further by many alternative approaches. Combined with the capabilities of up-scaling and unlimited mixing that spark ablation enables, this study provides an easy-to-use concept for producing the key building blocks for low-cost industrial-scale nanofabrication of advanced materials.

The ability to tailor engineering nanoparticles (ENPs) is a key to designing novel nanostructured materials for applications in printable electronics^1^, energy conversion and storage^2^,^3^, catalysis^4^,^5^, sensor technology^6^,^7^,^8^, as well as products in medicine^9^. The size and composition of ENPs are the two most important variables determining the properties of the resulting materials and products. Gas-phase methods for ENP synthesis exhibit fast kinetics because the relevant diffusion coefficients are three orders of magnitude larger compared to those encountered in wet-chemistry techniques. Although the fast kinetics enable effective continuous processes, which are gaining significant ground in view of industrial applications over the recent years^10^,^11^, they commonly lead to agglomerated particles which are undesirable in many cases.

Here we challenge the paradigm that “nanoparticle synthesis in the gas-phase leads to agglomerates”^12^ by coining a scalable concept of “singlet” particle production. Using spark ablation as an example of the vapour point source, we show that the concept can lead to singlets ranging from clusters of a few atoms to particles of any desired size by tuning the operating conditions. In addition, using first principles we derive an analytical model for predicting the size of the singlet particles under different operating conditions. The proposed approach exhibits enormous flexibility for high-throughput and ultrapure production^13^, thereby advancing ENP synthesis and enabling low-cost fabrication of nanomaterials on an industrial scale.

Traditionally, generation of ENPs is performed by wet-chemistry techniques, which offer unique possibilities of controlling particle shape^14^. These techniques, however, employ precursor solutions which commonly result in impurities on the synthesized ENPs as well as in hazardous wastes. In contrast to these classical paths, dry gas-phase methods provide more versatile and more environmentally friendly alternatives^15^, involving a very limited number of preparation steps, producing ENPs in a continuous manner, allowing for simple and continuous conditioning and deposition/immobilization, and generating very little wastes. Clean and simple gas-phase processes such as laser ablation^16^ and electric discharges^17^,^18^, can directly and locally vaporize bulk materials to form nanoparticles, thereby avoiding involvement of any additional compounds and guaranteeing high purity. The resulting ultrapure nanoparticless can be further processed in the gas-phase^19^,^20^,^21^,^22^, before being deposited and immobilized onto various (flat or porous) substrates (e.g., silicon wafers, glass slides, polymeric, filter membranes, ceramics), thereby opening a land of new possibilities for producing hierarchically patterned coatings and membranes^23^. Alternatively, nanoparticle synthesized in the gas-phase can be suspended into a liquid for coulpling to the wet-chemsitry routes, enabling other innovative methodes for novel material synthesis^20^,^24^,^25^,^26^.

Due to fast kinetics, continuous gas-phase methods generally yield agglomerates consisting of primary particles (typically regarded as the smallest size the particles can have) that are difficult to take apart^12^. A number of previous works have therefore focused on ways to avoid the collisions of these particles before deposition or immobilization. In spark discharges, for example, agglomeration can be reduced by using the high space charge density^27^, but this can be a limiting factor for the scalability of the technique. For flame aerosol synthesis, it has been shown that the associated elevated temperatures lead to non-agglomerated particles having sizes in the micron range^28^. Modifying the operating conditions in the gas-phase processes can also lead to the production of atomic clusters^29^,^30^,^31^.

In this report, we provide a general concept of continuous gas-phase synthesis of ultrapure singlet particles ranging from single atoms to particles in the nanometre range. The essence of this concept is illustrated in Fig. 1. Vapours are produced by localized material ablation using lasers or electric discharges. The vapours are strongly quenched by an inert gas flow of variable temperature, thereby producing particles by condensation. As the supersaturations reached in the rapidly quenched vapour cloud are extremely high, the critical nucleus size is pushed down to the atomic scale. As a result, the growth governed by particle-particle collisions can be considered to start from the atomic scale, and therefore particle-particle collisional growth represents a valid model for the description of the size distribution evolution^30^,^32^,^33^,^34^,^35^. Note that this simplification is only valid in the case of rapidly quenched vapours emitted from point sources. If the quenching flows are low (and thus the cooling rates are substantially low) as in most nanoparticle production methods in the gas phase, more sophisticated models will be required to describe particle formation and growth^36^. The atomic clusters and smallest nanoparticles that are formed at the early stages of the process are liquid-like even at room temperature^37^, and therefore fully coalesce into singlets when colliding with each other. Growth of singlets to a critical size above which coalescence only partly occurs or ceases for the selected operating temperature (see below), signals the onset of agglomeration which leads to non-spherical/agglomerated particles. For drastically quenched processes, the temperature in the particle growth phase can be decoupled from the localized vaporization, and can be set to a value guaranteeing complete coalescence (cf. Fig. 1). In contrast to other high temperature aerosol synthesis methods^28^,^38^, this feature provides great flexibility in controlling the size of the resulting nanoparticles.

## Results

### Theoretical framework

In order to exclusively produce singlet particles, the process must be controlled in a way that particle growth does not exceed the critical size, which in turn depends on the material of the particles and temperature^38^,^39^, and is relatively insensitive to other process parameters. In practice, singlets of any diameter can be achieved by controlling the temperature of the aerosol (i.e., particles dispersed within the carrier gas), so that coalescence is guaranteed up to the desired size. To further ensure that coalescence is not hindered by unwanted oxidation of particles due to the presence of trace amounts of oxygen and/or water in the carrier gas, extremely clean conditions are required throughout the production line. Such conditions can be achieved using suitable absorbers (i.e., molecular sieves and catalysts) to purify the carrier gas upstream the particle generator^40^.

In the next paragraphs we develop a simple model to predict the evolution of singlet nanoparticles produced by material ablation in the gas-phase under conditions that guarantee complete coalescence (see Supplementary Section S1). According to Smoluchoswski’s theory^32^, the decay rate of particle concentration d*N*(*t*)/d*t* is proportional to the square of *N*(*t*). Any particle losses by diffusional transport to the walls, where van der Waals forces normally guarantee sticking, can be approximated by a linear term in *N*(*t*)^41^,^42^. For sufficiently high concentrations during particle evolution, losses by diffusion to the walls as well as rapid turbulent dilution (cf. Supplementary Fig. S1 for additional details) can be neglected compared to the vigorous coagulation. *N*(*t*) can therefore be described by:


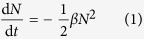


where 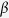 is the coagulation kernel, which changes with time depending on the momentary particle size distribution and temperature, gas flow conditions, and inter-particle forces^43^,^44^. For the early stages of atomic cluster formation, 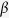 is unknown because the sticking probability after each collision depends largely on the stability of the atomic clusters^45^. Turbulent flow conditions that are not well enough defined in practical cases of particle coagulational growth, lead to yet another intractable problem in deriving any rigorous model for predicting the evolution of the particles. For tackling these problems, it is therefore instructive to evaluate the general behaviour of coagulating systems, governed by equation (1).

As the initial concentration *N*_0_ of the vapour atoms produced by material ablation is many orders of magnitude larger than the final concentration *N*(*t*_f_) of the particles (i.e., *N*_0_ >> *N*(*t*_f_)), equation (1) implies that *N*(*t*_f_) is independent of *N*_0_. In fact, *N*(*t*_f_) is only determined by the evolution of *N*(*t*) during approximately the last decade concentration reduction (cf. Supplementary Fig. S2)^46^. This stage, referred to as the “the final coagulation stage” in the following, covers most of the total coagulation time. Therefore, the complex turbulent flow conditions and the uncertainties in using an appropriate value for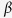 (associated with the early stages of atomic clusters growth) can be reasonably excluded as explained further in the supplement (cf. Supplementary Section S1 and S2 therein). For suitable ablation methods, the temperature in the particle growth region can be decoupled from that in the vaporization stage, and set to a well-defined value during the final coagulation stage. For a specific mass ablation rate, the particles grow approximately by a factor of two during the final coagulation stage due to the proportionality of particle size *d*_p_ to 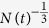. Coagulation models show that *β* does not vary by more than a factor of two when particle size doubles, and its dominating value corresponds to the final size. It is therefore reasonable to assume that *β* is constant and solve equation (1) considering *N*_0_ >> *N*(*t*_f_) to yield:


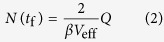


where *Q* is the gas volumetric flow rate and *V*_eff_ the effective volume corresponding to the volume incorporating most of the flow and occupied by the coagulating aerosol. In a continuous flow arrangement, the residence time of the particles is then *t*_f_ = *V*_eff_/*Q* and represents the duration for an aerosol parcel to travel from the vapour source to the point of measurement or further particle processing, where coagulation is inhibited by immobilization or adequate dilution. A good estimation for the relevant *β* can be based on the final particle size as explained above.

The increase of particle size due to coagulation is related to the decrease in number concentration. As the average particle size increased, the measured particle concentration drops while the total particle mass concentration 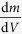 remains approximately constant^47^. Considering that the mass production rate 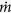 can be expressed as 
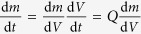
, the particle size *d*_p_ can be calculated by:


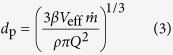


where 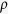 is the material density. This expression can be used to determine the particle size as a function of gas flow rate *Q* and particle mass production rate 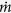. Equation (3) is generally valid for any material ablation process or similar processes where vaporization is localized enough for the coagulation temperature, defining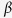, to be decoupled from the vaporization temperature, at least during the final coagulation stage. Evidently, rapid dilution (i.e., dilution time *t*_d_ << *t*_f_; cf. Supplementary Fig. S1) and high enough temperature for complete coalescence as mentioned above, must also be ensured.

Considering that the particle losses are neglected, equation (3) will generally lead to an overestimation of particle size. We expect, however, the effect of losses on particle size to be moderate for most systems because 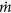 enters equation (3) as the cube root. We will show below that the model can be applied to a microsecond-pulsed particle source where in practice the system is mixed well enough to provide a uniform concentration during the final coagulation stage. The particle size predicted by equation (3) remains an approximation, but the elegance of the approach lies in the fact that uncertainties are circumvented because the final size depends on the final stage of the process, where the system is well defined.

From this point onwards we will consider spark ablation as the source of vapours for particle formation. As a scalable technique, spark ablation has, to the best of our knowledge, the greatest versatility of all nanoparticle production methods. Being applicable to many inorganic materials^13^, and virtually allowing unlimited mixing combinations at atomic and nanometre scale^48^,^49^, it represents a powerful approach for synthesizing advanced materials with multiple fuctionalities^50^,^51^.

Details of the spark ablation setup are described in the experimental section (cf. Supplementary Section S3 and Supplementary Fig. S3 for additional information). In brief, repeated microsecond-pulsed sparks initiated between two electrodes ablate electrode materials to produce vapour clouds. These vapours are subsequently quenched by a high-purity gas flow and condensed to form atomic clusters and nanoparticles. Depending on the process variables (i.e., quenching gas flow rate *Q*, spark energy *E*, and spark repetition frequencies *f*), the resulting particles can have sizes that range from clusters of a few atoms up to any desired size. For the sake of completeness, it must be noted here that if agglomerates are produced by spark ablation, or by any other similar gas-phase method, they can be converted to singlet particles by heating in gas suspension after growth has essentially ceased^38^,^52^,^53^. This technique has been applied in diluted laboratory setups with particle mass production rates in the range of mg h^−1^. By contrast, the concept presented in this work, where coalescence is induced in the particle growth phase, is only limited by the vapour mass production rate from the spark ablated electrodes. Considering that the measurements of ablated mass per spark indicate that a production rate of the order of 1 g h^−1^ is feasible (cf. Supplementary Section S4), the concept can lead up to three orders of magnitude higher singlet particle production rate than other commonly used techniques^13^. Additionally, the possibility of numbering up the generators can further increase the production rate of the desired particles to meet industrial demands^10^.

The mass produced by a single spark is given by:


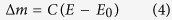


here *C* is a material-dependent constant (cf. Supplementary equation (S6)), *E* the spark energy, and *E*_0_ is the minimum spark energy for producing particles (cf. Supplementary equation (S7)). *C* and *E*_0_ can be calculated by the evaporation model, which is derived by the energy balance of the evaporation process (cf. Supplementary equation (S5))^54^. The mass production rate is given by 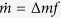 (cf. Supplementary Fig. S4), where *f* is the spark repetition frequency. Pulsed sparks can be regarded as a continuous particle source given that sufficient mixing guarantees a uniform concentration before the final coagulation stage is reached. Combining equations (3) and (4) yields:


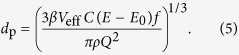


Recipes and sources for determining sufficiently accurate values for the required quantities of *C*, *E*_0_, *β*, and *V*_eff_ are given in the supplement (cf. Supplementary Section S4 and S5). In the following, we justify the proposed model (i.e., equations (2) and (3) that give the evolution of particle concentration and size) with the measurements.

### Experimental validation

**Figure 2:**
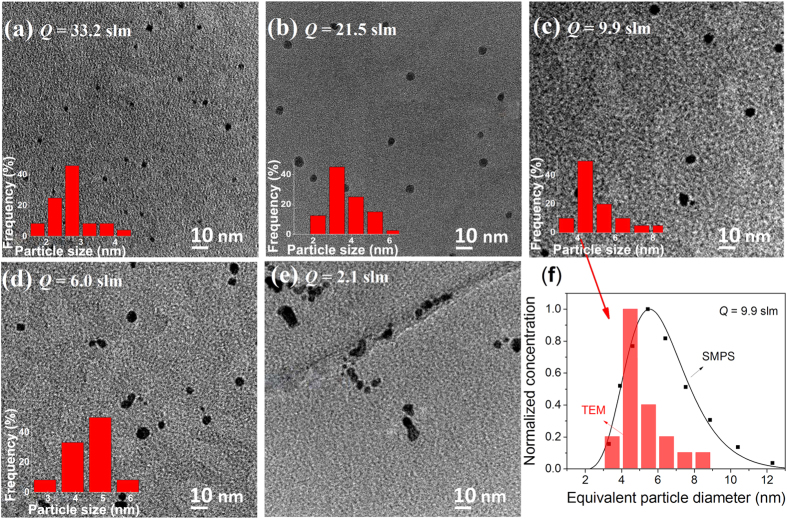
Electron Micrographs of Au singlets(a–c) and partly agglomerated particles (d and e) produced by spark ablation, and particle size distributions determined by the SMPS and TEM image at *Q *= 9.9 slm (f). (d,e) show that the size of primary particle is ca. 6 nm, which corresponds to the largest singelt particles of Au.

Figure 2(a–e) shows TEM images of particles collected using our experimental setup (cf. Supplementary Section S3 and Supplementary Fig. S3) under different quenching gas flow rates at a fixed spark energy *E* and frequency *f*, which allows variation of the singlet particle size from ca. 3 nm upwards. Evidently, the coalescence is complete up to sizes between 5 and 6 nm (cf. Supplementary Fig. S6 for more details). Larger particles are agglomerates, indicating that the selected coagulation temperature of ca. 20 °C is adequate for generation of Au singlet particles up to ca. 6 nm.

In coagulation with full coalescence, the size distribution approaches a lognormal self-preserving distribution with geometric standard deviation (GSD) ranging from ca. 1.33 to 1.35^55^. Figure 2f shows that the geometrical mean diameter (GMD) of the singlet particles measured by the scanning mobility particle sizer (SMPS) is ca.10% larger than that determined by the TEM images when *Q* = 9.9 standard litres per minute (slm). This discrepancy can be explained by the larger representation of the small particles (having large diffusion coefficients) collected by diffusion on the TEM grids. The limited number of particles counted in TEM images also adds a minor statistical error in the microscopy observations.

**Figure 3:**
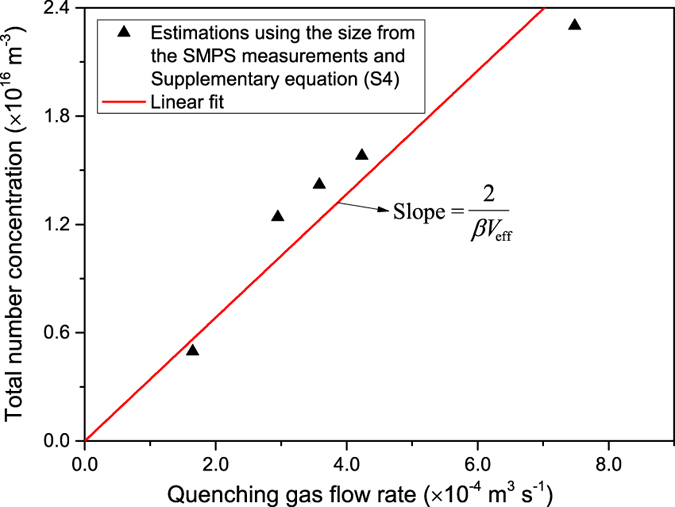
Concentration of Au singlet particles as a function of quenching gas flow rate at a spark energy of 16 mJ and a spark repetition frequency of 60 Hz.

Figure 3 shows how the total number concentration of the particles at the exit of the particle generator varies with quenching gas flow rate. It should be noted here that the concentration is derived from the particle sizes measured by the SMPS with the assumption that there are no diffusional losses, i.e., mass of ablated material per unit volume is conserved (cf. Supplementary Fig. S2 and Supplementary equation (S4)). It should be pointed out that in order to derive the particle size distributions at the exit of the particle generator, the space charge effect in the differential mobility analyser (DMA)^56^ is neglected as it affects the particle size measurements by less than 4% assuming the highest possible concentration of charged particles we observed (ca. 10^7^ cm^−3^; cf. Supplementary Section S7). Moreover, for particle concentrations of the order of 10^10^ cm^−3^, coagulation within the DMA can also be neglected during the short residence time of 0.15 s, as discussed in Supplementary Section S2 (cf. Supplementary Fig. S2).

The estimation of singlet particle concentration confirms the dependence of *N*(*t*_f_) on *Q* predicted by equation (2), which has been derived using a constant 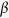 and assuming that *N*_0_ >> *N*(*t*_f_). The slope (i.e., 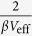) of the best-fitted straight line through the measurements indicates that the product 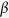
*V*_eff_ is 5.93 × 10^−20^ m^6^ s^−1^. This value is in line with a theoretical value for 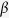 that considers poly-dispersity as well as van der Waals forces between the particles (cf. Supplementary Section S5.1 and S5.2)^44^,^57^. Note that the image potential described by Ouyang *et al.*^44^, can be neglected for our low concentration of charged particles (cf. Supplementary Section S5.3). The value of 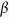 is in the order of 10^−16^ m^3^ s^−1^ (cf. Supplementary equation (S8)) based on the final (desired) size and an estimated effective volume *V*_eff_ of 10^−4^ m^3^ corresponding to an adequate fraction of the volume of our spark chamber (cf. Supplementary Fig. S5).

**Figure 4:**
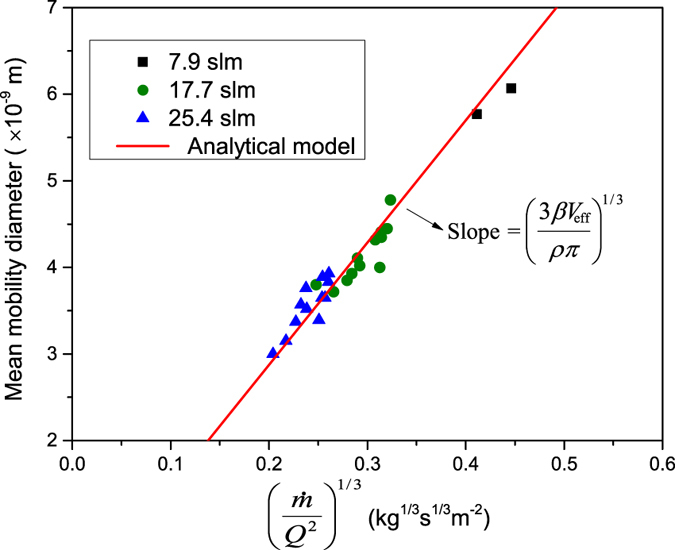
Mean mobility diameter of particles produce by spark ablation as a function of mass production rate and quenching gas flow rate.

Figure 4 shows the variation of the GMD with the term 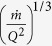, which is linear according to equation (3). The slope (i.e., 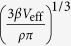) of the best-fitted solid line through the experiments is 1.42 × 10^−9^ kg^−1/3^ s^−1/3^ m^3^. For a density of 19.3 g cm^−3^ for Au, the product 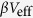 derived from this slope is 5.78 × 10^−20^ m^6^ s^−1^, which is in good agreement with the value derived from Fig. 3, and thus also with the theoretical value of 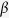 and an estimate of 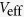 from the geometry of the confinement (see details in Supplementary Section S5). The value of 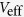 has the highest uncertainty. For systems of similar geometry to that used in this work we recommend 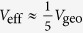, where 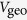 is the geometric volume of the confinement (details are provided in Supplementary Fig. S5). Note that an error in 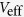 within a factor of two would only alter the predicted *d*_p_ by 25%. Only the experimental values for particles having sizes below 7 nm are plotted in Fig. 4, since larger particles are agglomerates (cf. Fig. 2e) due to incomplete coalescence.

## Discussion

In principle, the GMD of singlet particles produced via spark ablation (and any other similar gas-phase process) can be tuned from atomic clusters to any desired size by carefully varying the gas flow rate *Q*, and the mass production rate 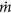, which in turn is defined by the spark energy *E* and the spark repetition frequency *f.* Of course, full coalescence must be guaranteed by choosing a sufficiently high operating temperature and a high-purity carrier gas. In order to up-scale the singlet ENP production process, one needs to increase the production rate 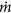. This can be achieved by numbering up the particle generators, with each of them delivering a mass rate in the order of 1 g h^−1^.

Our model can estimate the singlet size distribution at the outlet of the nanoparticle generator used in this work. The GMD is given by equation (3) while the lognormal size distribution has a self-preserving GSD of ca. 1.35^55^. Mobility size classification in the gas-phase can easily be applied for applications requiring narrower size distributions^58^, although it should be noted that doing so will lead to substantial particle losses and will therefore limit scalability.

Deposition of well-defined singlets on a substrate can yield films and materials of unique properties. Avoiding coalescence of the colliding particles on the substrate is a prerequisite to retain these properties. An elegant way to hinder coalescence is by coating the particles (e.g., with an oxidized layer) while they are still suspended in the gas (i.e., before deposition)^59^,^60^. For deposited uncoated particles, coalescence on the substrate can be avoided by keeping the surface coverage low enough (cf. Supplementary Fig. S8) or by manipulating charge effects between particles^27^,^61^. Low surface coverages are sufficient to improve the conversion efficiency of solar cells and photo catalysts for water splitting^62^,^63^. In the case of high surface coverage, where the particles are in contact, the substrate temperature should be controlled below the threshold temperature to avoid the coalescence of the arriving singlets^64^,^65^. Integrating gas-phase synthesis of singlets to wet-chemistry routes can also be used for supressing particle collisions, since the collision rate is decreased by three orders of magnitude in the liquid phase, and opens numerous possibilities of further processing^20^.

In summary, we introduce a general concept for continuous gas-phase synthesis of well-defined singlet particles in the nanometre size regime and even below that. The concept of promoting coalescence by using an ultrapure carrier gas and a sufficiently high temperature in the particle growth zone, has been tested on the example of synthesizing Au nanoparticles smaller than ca. 6 nm using spark ablation. In addition, we derive an analytical model that can be used to determine the combination of process parameters required to obtain singlet nanoparticles of a desired size. The model can be applied to predict the size (ranging from that of single atoms to any value) of singlet nanoparticles (consisting of any material; cf. Supplementary Fig. S9 of the Ag singlets) produced by rapidly quenched gas-phase processes.

Combined with the various advantages of continuous gas-phase processes, including their scalability, high particle purity and high versatility (i.e., particles of virtually any inorganic composition or mixture that spark ablation enables), the method used as an example here (spark ablation) exhibits enormous flexibility for high-throughput production of ultrapure singlets, especially in the size regime below 10 nm where other continuous scalable methods hardly exist. Consequently, the technique enables the advancment of ENP synthesis and paves the way towards cost-effective fabrication of novel nanomaterials for numerous applications (cf. Supplementary Section S8) on the industrial scale.

## Methods

### Spark Discharge Generator

A spark discharge generator consists of a pair of electrodes with a gap of ca. 1 mm between them, connected to an electric circuit (see Supplementary Fig. S3). The circuit induces microsecond pulsed discharges in a typical range of energy per spark from 0.3 to 200 mJ and a repetition frequency ranging from 0.1 to 25 kHz^13^. An inert gas flow continuously flushes the inter-electrode gap carrying away the produced vapors and particles to the point where they can be processed.

### Experimental Set-up

The system consists of units for the generation (I), collection (II), and online size distribution measurement (III) of nanoparticles (cf. Supplementary Fig. S3). The size distributions of the resulting aerosols are measured with a Scanning Mobility Particle Sizer (SMPS) system, consisting of a differential mobility analyzer (DMA), and a Faraday cup aerosol electrometer (AEM), but avoiding an aerosol neutralizer. The singlet nanoparticless produced by the spark ablation were collected on TEM grids, using a custom-made electrostatic precipitator (ESP) placed at the DMA outlet. The DMA classifies the particles according to their mobility. The resulting nanoparticles are led to the SMPS through a ca. 0.4-m long stainless steel tube with an inner diameter of 4 mm.

## Additional Information

**How to cite this article**: Feng, J. *et al.* Toward industrial scale synthesis of ultrapure singlet nanoparticles with controllable sizes in a continuous gas-phase process. *Sci. Rep.* **5**, 15788; doi: 10.1038/srep15788 (2015).

## Supplementary Material

Supplementary Information

## Figures and Tables

**Figure 1:**
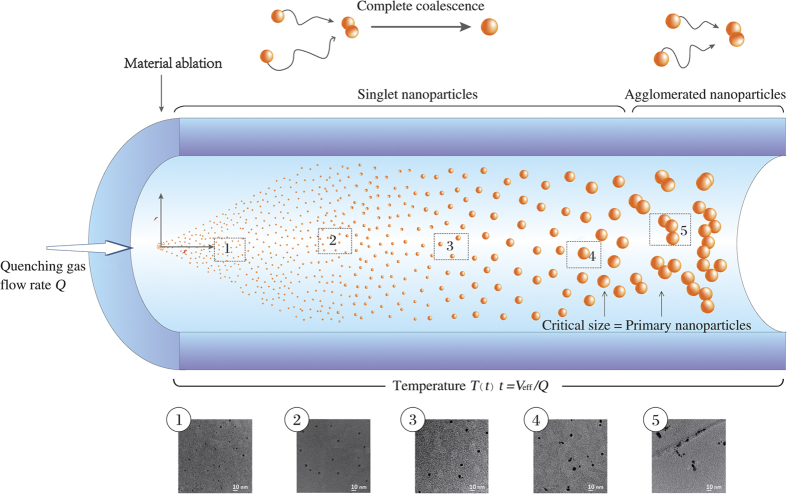
Schematic representation of the formation of singlet and agglomerated aerosol nanoparticles resulting from material ablation at atmospheric pressure.
