# Supplementary material for: Toward industrial scale synthesis of ultrapure singlet nanoparticles with controllable sizes in a continuous gas-phase process
Source: Sci Rep. 2015 Oct 29;5:15788. doi: 10.1038/srep15788 (PMC4625176; doi:10.1038/srep15788)
Supplement: Supplementary Information [file srep15788-s1.pdf]

www.nature.com/scientificreports

SCIENTIFIC REPORTS

Supplementary Information

Toward industrial scale synthesis of ultrapure singlet nanoparticles with controllable sizes in a continuous gas-phase process

Jicheng Feng[1], George Biskos[1, 2, 3, 4], and Andreas Schmidt-Ott*[,1]

[1]Department of Chemical Engineering, Delft University of Technology, Delft, 2628 BL, The Netherlands

[2]Faculty of Civil Engineering and Geosciences, Delft University of Technology, 2628 CN, Delft, The Netherlands

[3]Energy Environment and Water Research Centre, The Cyprus Institute, Nicosia 1645, Cyprus

[4]Department of Environment, University of the Aegean, Mytilene 81100, Greece

[*]To whom correspondence should be addressed: A.Schmidt-ott@tudelft.nl

1. Material ablation in continuous gas-phase

Localized material ablation guarantees a high purity of the resulting nanoparticles, and allows decoupling of the high temperature required for vaporization from the system temperature $T(t)$ where particle growth takes place[1–3]. This advantageous feature allows that the system temperature $T(t)$ can be regulated in order to ensure full coalescence of colliding particles to any desired size.

Another important feature of ablation processes is that they yield an initial particle or vapor atom concentration N_0 that is several orders of magnitude higher than the final particle concentration $N(t_f)$, i.e., $N_0 >> N(t_f)$[4]. This feature allows for analytical estimation of particle concentration evolution by excluding uncertainties at the early stage of particle formation process (*e.g.*, sticking probability, turbulence).

The quenching gas flowing through the electrode gap causes turbulent dilution of the initial vapours and particles. The time period t_d that the vapour atoms and particles spend in this dilution stage is much shorter than the total period of particle growth and thus can be reasonably neglected as discussed in Supplementary Section 1.1 below.

1.1 Turbulent dilution time t_d

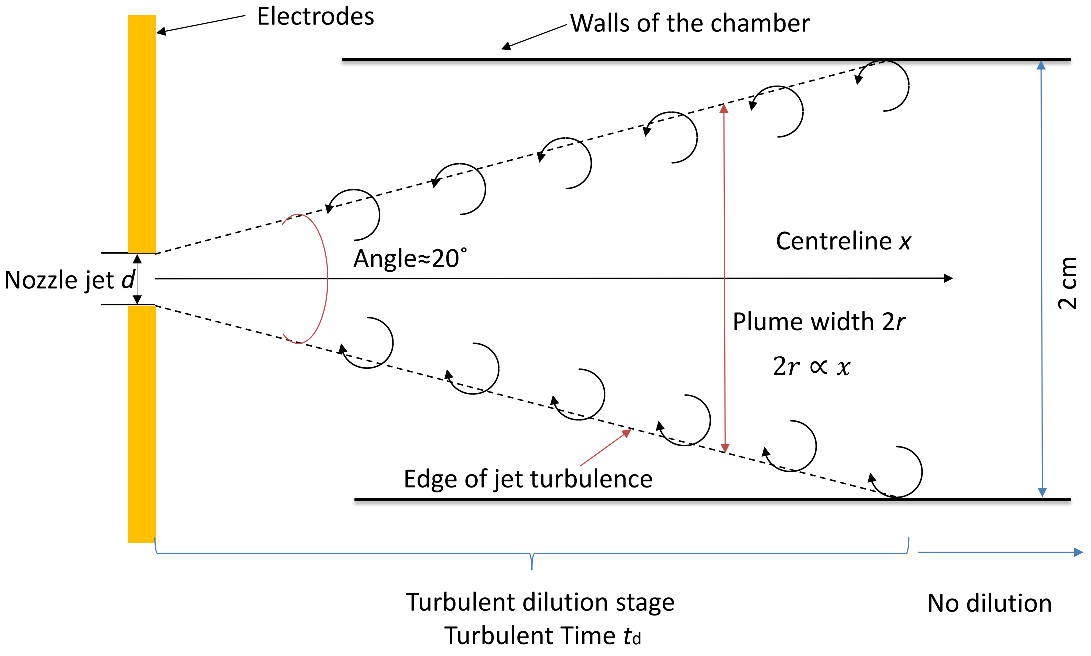

Supplementary Figure S1. Illustration of the flow structure in the turbulent dilution and the convective region immediately downstream the spark discharge.

The flow downstream the spark ablation can be split into two regions: the first that exhibits turbulent dilution and the second where particles reach the tube walls. To understand which

process is more dominating we need get an estimate of the residence time of the particles in each of these two regions. Here we use the simplest description that assuming the jet region flares out linearly in the x direction. The time-averaged turbulent flow pattern is a conical volume with an angle of roughly 20°[5]. In the time-averaged turbulent jet, x is measured from the virtual origin of the cone and is proportional to the plume width $2r$ ($\mathrm{d}r/\mathrm{d}x \approx \tan(10°)$). The plume is developing before its boundaries reach the walls of the spark chamber. The inner diameter of the spark chamber is about 2 cm as shown below.

The travelling distance of the particles flowing through the jet is estimated as $L = r/\tan(10°) \approx$ 5.7 cm, and the time of travel along the x direction is obtained by $\mathrm{d}t = \mathrm{d}x/u_\mathrm{a}$. u_a is the time-averaged fluid speed at the centreline given by[5]:

$$u_\mathrm{a} = U_0\left(\frac{x}{x_0}\right)^{-1/2} \qquad (S1)$$

where U_0 is the mean fluid velocity at the nozzle and x_0 is given by[5]:

$$x_0 = \frac{3}{4}\gamma d \qquad (S2)$$

where d is the nozzle diameter, $\gamma = 7.67$ the empirical constant accounting for the growth rate of the jet region. Combining Supplementary equation (S1), (S2) and $\mathrm{d}t = \mathrm{d}x/u_\mathrm{a}$ yields $\mathrm{d}t = \frac{\left(\frac{x}{x_0}\right)^{1/2}}{U_0}\mathrm{d}x$ which can integrate for 0 to t_d and for x_0 to L the left and right hand of the equation, respectively, yields:

$$t_\mathrm{d} = \frac{2}{3U_0{x_0}^{1/2}}\left(L^{3/2} - {x_0}^{3/2}\right) \qquad (S3)$$

Using a quenching gas flow rate of 10 standard litres per minute (slm), the turbulent time is t_d = 2.5 ms. In contrast, the total residence time of particles in our apparatus is in the order of 1 s. Hence, the turbulent dilution time can be reasonably neglected. This further justifies the assumption of mass density conservation in the total period of particle growth.

2. Final coagulation stage

Supplementary Figure S2 shows the evolution of the number concentration predicted by equation (1) of the main manuscript with time due to poly-disperse coagulation. In the case of the straight line on the logarithmic plot marked "$N_0 \to \infty$" was calculated using equation (2) of the main text assuming a constant coagulation kernel β calculated by the final particle size.

The residence time V_{eff}/Q in equation (2) is simply replaced by the residence time t. Note that β has been enhanced by a self-preserving size distribution with respect to mono-disperse case. Given that the self-preserving value of the geometric standard deviation (GSD) is reached approximately after the time of the first reduction of the concentration by a factor of 10, we can also apply equation (2) for the case of arbitrary size distributions[6], if we account for the enhancement factor of a certain poly-dispersity for β with respect to the mono-disperse case[7]. At the moment the curves for different initial concentrations reach this straight line (i.e., $N_0 \to \infty$), the concentration becomes independent of the initial concentration.

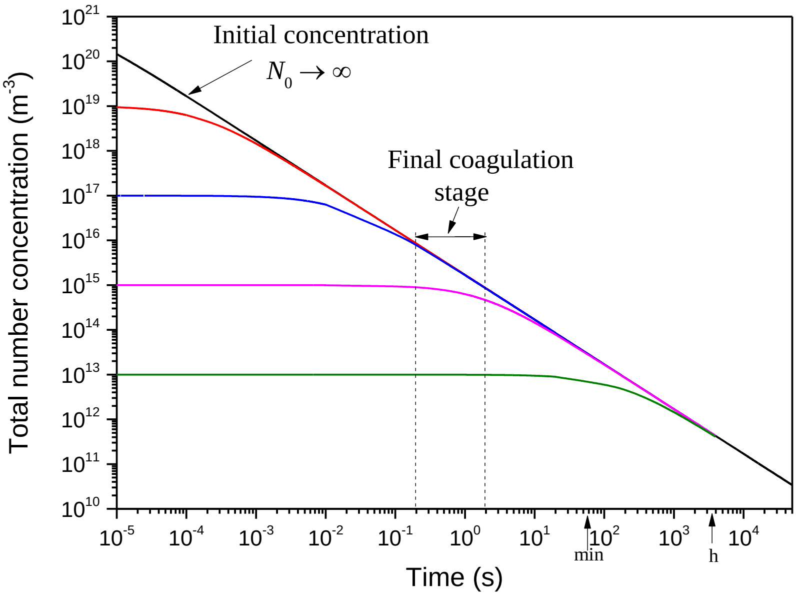

Supplementary Figure S2. Particle number concentration versus time for various initial concentrations N_0. The constant coagulation kernel β used in these predictions accounts for poly-dispersity (i.e., self-preserving distribution) that increases the final particle size as compared to the mono-disperse coagulation (cf. Supplementary equation (S8) and Supplementary Section S5).

However, β depends on the particle size. For a specific mass ablation rate, the particles grow approximately by a factor of 2 during the final coagulation stage due to the proportionality of particle size d_p with $N(t)^{-\frac{1}{3}}$. Because β does not vary more than a factor of 2 in the final coagulation stage, and its dominating value corresponds to the final size, we can assume that β is constant. Although assuming a constant β of a final particle size remains an approximation, Supplementary Figure S2 illustrates a general feature: any coagulation process approaches a state where the particle concentration is independent of its initial value. This would not be any difference, if β spreads out to have high-variability with size, only the line for "$N_0 \to \infty$" is

curved for this case. Regarding the poly-disperse coagulation, we can regard the end of the turbulent dilution period as the initial state.

If the general concentration evolution of self-preserving size distribution according to Supplementary Fig. S2 is applied to our case, the time interval between 0.2 to 2 s is marked in the figure. It is seen that $N(t_f)$ evolves into the same line of $N_0 \rightarrow \infty$ regardless of its initial concentration, if only $N_0 \geq 10^{17}$ m^{-3}. It also becomes clear that the concentration $N(t_f)$ is mostly determined by the evolution of $N(t)$ during approximately the last decade of N-reduction or the last decade in time.

3. Experimental materials

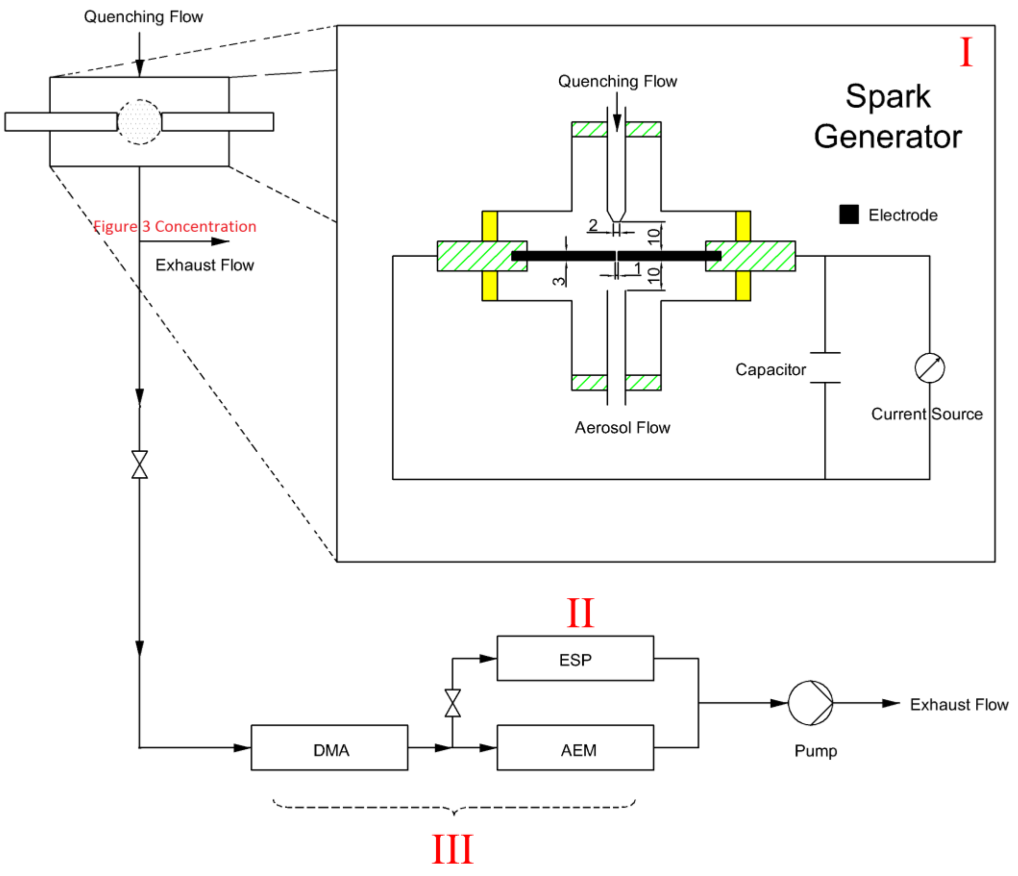

Supplementary Figure S3. Schematic diagram of the experimental setup. All dimensions are expressed in mm. Key: DMA, differential mobility analyzer; ESP, electrostatic precipitator; AEM, aerosol electrometer.

Supplementary Figure S3 shows the details of the experimental setup described in the Methods section of the main manuscript. The system consists of components for the generation (I), collection (II), and online size distribution measurements (III) of nanoparticles. Some acronyms are shown in the following subsection. The electrode rods (99.99% purity,

Au and Ag, 3 mm diameter, 25 mm long) used in the spark generator were purchased from MaTecK Material-Technologie Kristalle GmbH. For all experiments discussed in this paper, N_2 (purity 99.999%) is used as a carrier gas.

The measured concentrations plotted in Figure 3 are derived from the geometric mean particle diameter measured by SMPS and assuming negligible wall losses, i.e., mass conservation per unit volume. The total particle concentration is therefore given by:

$$N_{\mathrm{tot}} = \frac{6\dot{m}}{\rho {d_{\mathrm{p}}}^{3} Q} \qquad (S4)$$

While particle size can reliably be determined by means of the SMPS, this is not the case for the absolute particle concentration, as explained below. An SMPS system, generally consists of a bipolar charger (also called “neutralizer”), a DMA and an AEM. However, a neutralizer would increase the residence time of the nanoparticles before entering the DMA by ca. 7 s. This long residence time leads to strong coagulation and agglomerate formation, which we want to avoid in the present study. Another reason for not using the bipolar charger is that it would not give us any additional information given that a fraction of particle produced by spark ablation is already bipolarly charged. At the high particle concentration we have, a bipolar charger would not deliver the well-defined equilibrium charge distribution which would be necessary for an absolute particle concentration measurement[8], providing another reason to avoid using it.

The bipolar charger is frequently omitted by people routinely characterizing particles produced by spark production. Tabrizi *et al*. have shown that the total particle size distribution can be represented by the charged particles directly from the spark discharge in a relative sense[9], which means that the mean sizes are correctly reflected. This can be explained by the fact that spark ablation produces a high concentration of ions of both polarities similarly to the bipolar charger. For small particles, it can easily be shown that this rule is even valid if charge equilibrium is not reached. It can also be shown that coagulational growth of these small particles alters the size distribution of the neutral particles and the charged particles in a very similar way. The detailed explanations for the aforementioned points are described in the next paragraph.

Our measurement-based values for the charged particle concentration and the total particle concentration imply that the particle collision frequency of the charged particles among each other, leading to recombination and thus loss of charges, must be negligible during the period

between completion of charging near the spark and measurement. Thus the charged particles retain their charge but grow in size by colliding with neutral particles. Originally, the relative charging probabilities corresponding to different sizes within the self-preserving distribution are proportional to d_p^2,[10] which would lead to the correct derivation of the total particle size distribution by the SMPS system regarding the position of the geometric mean size (see above). The growth of the geometric mean size of this self-preserving distribution by a factor c_{sp} retains the said proportionality with d_p^2, because each size within the self-preserving distribution grows by the same factor (this makes it self-preserving). So the particles of a size d_{p1} grow to the size $d_{1g} = c_{sp}d_{p1}$, and the particles of a size d_{p2} grow to the size $d_{2g} = c_{sp}\ d_{p2}$. The corresponding initial charging probabilities $P_1 \propto d_{p1}^2$ and $P_2 \propto d_{p2}^2$ remain unchanged after growth[10], thus $P_{1g} \propto d_{p1}^2$ and $P_{2g} \propto d_{p2}^2$. The SMPS system assumes $P_{1g} \propto c_{sp}^{\ 2}\ d_{p1}^2$ and $P_{2g} \propto c_{sp}^2\ d_{p2}^2$, but the ratio P_{1g}/P_{2g} remains constant, which means that the SMPS system indicates a distribution that correctly represents the geometric mean size but underestimates the total particle number concentration.

3.1 Characterization methods

3.1.1 Online characterization

The DMA selects particles based on their different sizes, whereas the AEM measures the current of all the singly charged particles downstream the DMA. This current, which depends on the aerosol flow rate used in the measurements, defines the concentration of charged particles of the polarity chosen by the DMA (negative in our case). Note that a bipolar charger typically used in SMPS system is not employed in our measurements. One reason is that the spark produced particles are already bipolarly charged and represent the total particle size distributions as explained above[9]. The other reason is that the longer residence time (ca. 7 s) inside a bipolar charger would have led to considerable agglomeration, which we want to avoid in the present study.

3.1.2 Offline characterization

Transmission electron microscopy (TEM) and scanning electron microscopy (SEM) were used to image the particles produced by the spark generator. An electrostatic precipitator (ESP) was used to collect the charged particles produced by the spark generator and classified by the DMA. TEM grids (Van Loenen Instruments, S143-3 Q'foil 1.2/1.3 400 Cu) and substrates covered by TiO_2 layer were inserted into the ESP. The DMA voltage is fixed at the peak of the particle size distribution.

4. Ablated mass per spark

Equation (4) from the main manuscript is expanded below. The mass ablated from one spark can be estimated by the Llewellyn Jones model given by[11]:

$$\Delta m = \frac{\alpha E - 2\pi r^2 \sigma \tau (T_b^4 - T^4) - 2\pi r \tau k_{\mathrm{e}} (T_{\mathrm{b}} - T) - 2\pi r \tau k_{\mathrm{a}} (T_{\mathrm{b}} - T)}{c_{\mathrm{ps}} (T_m - T) + c_{\mathrm{pl}} (T_{\mathrm{b}} - T_{\mathrm{m}}) + H_{\mathrm{m}} + H_{\mathrm{e}}} \qquad (S5)$$

where E is the energy per spark (J); α is the fraction of spark energy used for evaporation; c_{ps} and c_{pl} (J K^{-1} kg^{-1}) are the heat capacities of the solid and liquid material, respectively; τ (s) is the spark duration; r (m) is the radius of the spark channel; k_{e} and k_{a} (W m^{-1} K^{-1}) are the thermal conductivity of the electrode material and carrier gas (N_2, $k_{\mathrm{a}} = 25.83 \times 10^{-3}$ W m^{-1} K^{-1}), respectively; T, T_{b}, and T_{m} (K) are the carrier gas temperature at 293 K (e.g., room temperature), the boiling and melting points of the electrode materials, respectively; H_{m} and H_{e} (J kg^{-1}) are the enthalpies of melting and vaporization of electrode materials; and σ (5.67×10^{-8} W m^{-2} K^{-4}) is the Stefan–Boltzmann constant. Values of all material properties (for Au and Ag) are given in Supplementary Table S1.

Supplementary Table S1. Physical constants at atmospheric conditions

Parameters	Au	Ag
c_{ps} (J K^{-1} kg^{-1})	129	235
c_{pl} (J K^{-1} kg^{-1})	129	235
k_{e} (W m^{-1} K^{-1})	318	429
T_{b} (K)	3243	2435
T_{m} (K)	1337	1235
H_{m} (J kg^{-1})	6.37×10^4	1.04×10^5
H_{e} (J kg^{-1})	1.74×10^6	2.35×10^6

Supplementary Figure S4 shows measurements of the mass ablation by one spark from the electrodes as a function of the spark energies. The result is consistent with proportionality between Δm and (E-E_0). This justifies the fraction of energy α consumed for production of nanoparticles is constant for identical gap distances. Note that the value of α is validated for all materials[9]. Threshold energy E_0 and the slope C are derived from the fitted straight line through the experiments shown in Supplementary Fig. S4.

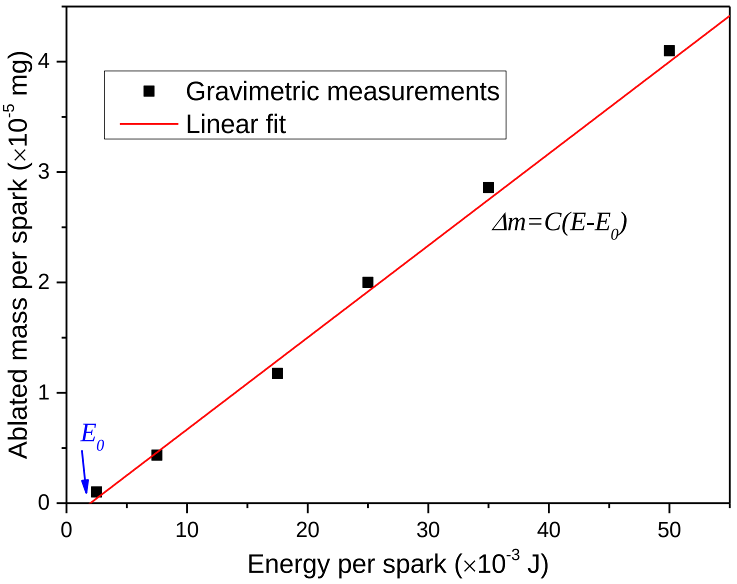

Supplementary Figure S4. Mass ablated from the electrodes by one spark as a function of the spark energy

---

The material-dependent constant C is given by:

$$C = \frac{\alpha}{c_{\text{ps}}(T_{\text{m}} - T) + c_{\text{pl}}(T_{\text{b}} - T_{\text{m}}) + H_{\text{m}} + H_{\text{e}}} \qquad \text{(S6)}$$

Its value is derived as 8.33×10^{-10} kg J^{-1} when α=0.1819% from the slope of the straight line in Supplementary Fig. S4, which can be considered independent of the electrode materials for identical spark gaps[9,11].

The threshold energy E_0 to produce nanoparticles via spark ablation is:

$$E_0 = \frac{2\pi r^2 \sigma\tau(T_{\text{b}}^4 - T^4) + 2\pi r\tau k_{\text{e}}(T_{\text{b}} - T) + 2\pi r\tau k_{\text{a}}(T_{\text{b}} - T)}{\alpha} \qquad \text{(S7)}$$

Assuming that the radius of spark ablated hot-spot r is 0.51 μm and the spark duration τ is 1.2 μs, Supplementary equation S6 yields E_0 = 1.98 mJ. This value is in agreement, within experimental uncertainty, with the estimated value of 1.97 mJ by the fitted line through the experiments shown in Supplementary Fig. S4.

A newly developed switching circuit that delivers high frequency sparks ranging from 1 to 25 kHz described in our previous publication[12]. Based on the data in Supplementary Figure S4 at spark energy of 50 mJ and 25 kHz repetition frequency, the mass production rate $\dot{m} = \Delta m f$ of single synthesis unit can go up to 25 kHz × 4 × 10^{-5} mg × 3600 s = 3.6 g/h. Arrays of these units could be used to scale up production to any desired rate.

5. Enhancement of the coagulation

In this section, we discuss the enhancement of coagulation by both poly-dispersity, van der Waals forces between particles and image potentials between net neutral nanoparticles and charged ones based on the Fuchs' interpolation model for mono-disperse particles.

5.1 Mono-disperse coagulation

Fuchs' coagulation theory[13], gives the Brownian coagulation kernel β for particles of equal size as follows:

$$\beta = 8\pi D d_{\mathrm{p}} \left(\frac{d_{\mathrm{p}}}{d_{\mathrm{p}} + g} + \frac{8D}{c d_{\mathrm{p}}} \right)^{-1} \quad \text{(S8)}$$

Here D is the particle diffusion coefficient, g the transition parameter, and c a function of temperature and the mass of colliding particles (cf. Supplementary Table S2). Supplementary equation (S8) assumes sticking at every collision and no forces between the particles. Because $N(t_{\mathrm{f}})$ is sensitive only to the value of β in the final coagulation stage as discussed above, we can use Supplementary equation (S8) to estimate the constant value used in our model by using the desired particle size. For 5 nm particles, β is 3.29×10^{-16} m^3 s^{-1}.

5.2 Poly-disperse enhanced coagulation

Apart from the particle size, the coagulation rate depends on the broadness of the size distribution and inter-particle forces. The coagulation rate for aerosols having a log-normal size distribution is enhanced with respect to the mono-disperse case, so a correction factor has to be used to predict the resulting size distribution more accurately[7,14]. The fact that a coagulating aerosol rapidly approaches a self-preserving size distribution can be directly used to most cases, as the self-preserving distribution is certainly reached in the final coagulation stage (as indicated by the size distribution measurements shown in Fig. 2f in the main manuscript), which is crucial for $N(t_{\mathrm{f}})$ and d_{p}. Based on this fact and assuming a singlet particle diameter of 5 nm, we estimate that $\beta = 3.94\times10^{-16}$ m^3 s^{-1} [7], which is ca. 20% larger than the value given by Supplementary equation (S8).

5.3 van der Waals forces enhanced coagulation

Although previous studies have showed that the majority of particles produced by spark ablation is uncharged[9], ignoring inter-particle forces leads to inaccurate predictions of coagulational growth. For metal particles in the free molecular regime, the effect of van der

Waals forces on coagulation is significantly pronounced[15]. For example gold particles in the size range of 1~20 nm require an enhancement factor of ca. 2.2 to match observations with predictions[15–17]. We directly apply this factor to the particle size distribution shown in Figure 2f and Figure 4 of the main manuscript. The coagulation kernel enhanced by van der Waals forces then increases to $\beta = 8.67\times10^{-16}$ m^3 s^{-1}.

Supplementary Table S2. Fuchs form of the Coagulation Kernel β

Coagulation Kernel[13]	$\beta = 8\pi D d_\mathrm{p}\left(\frac{d_\mathrm{p}}{d_\mathrm{p}+g}+\frac{8D}{c d_\mathrm{p}}\right)^{-1}$
Diffusion coefficient[14]	$D = \frac{kTC_\mathrm{c}}{3\pi\mu d_\mathrm{p}}$
Cunningham slip correction factor[14]	$C_\mathrm{c} = 1 + \frac{\lambda}{d_\mathrm{p}}(2.34 + 1.05\exp(-0.39\frac{d_\mathrm{p}}{\lambda})$
Particle velocity [13]	$c = (\frac{8kT}{\pi m})^{1/2}$
Transition parameter [13]	$g = \frac{1}{3d_\mathrm{p}l}[(d_\mathrm{p}+l)^3 - (d_\mathrm{p}{}^2 + l^2)^{3/2}] - d_\mathrm{p}$ $l = \frac{8D}{\pi c}$

5.3 Image potential enhancement

Image potential is frequently the dominant factor of potential energy in collisions between net neutral particles and charged particles[15]. Assuming that the concentration of the charged particles is ca. 1% of the total particle number concentration, the image potential term is only 2% of the concentration decay rate determined by equation (1), and can therefore be neglected for even lower charge particle fraction in this work (considerably lower than the equilibrium state).

Dividing βV_eff derived from the data shown in Fig. 3 and Fig. 4 by this value, the estimated effective volume V_eff is around 66 cm^3, which is 1/5 of the total geometric volume of the spark chamber (cf. Supplementary Figure S5).

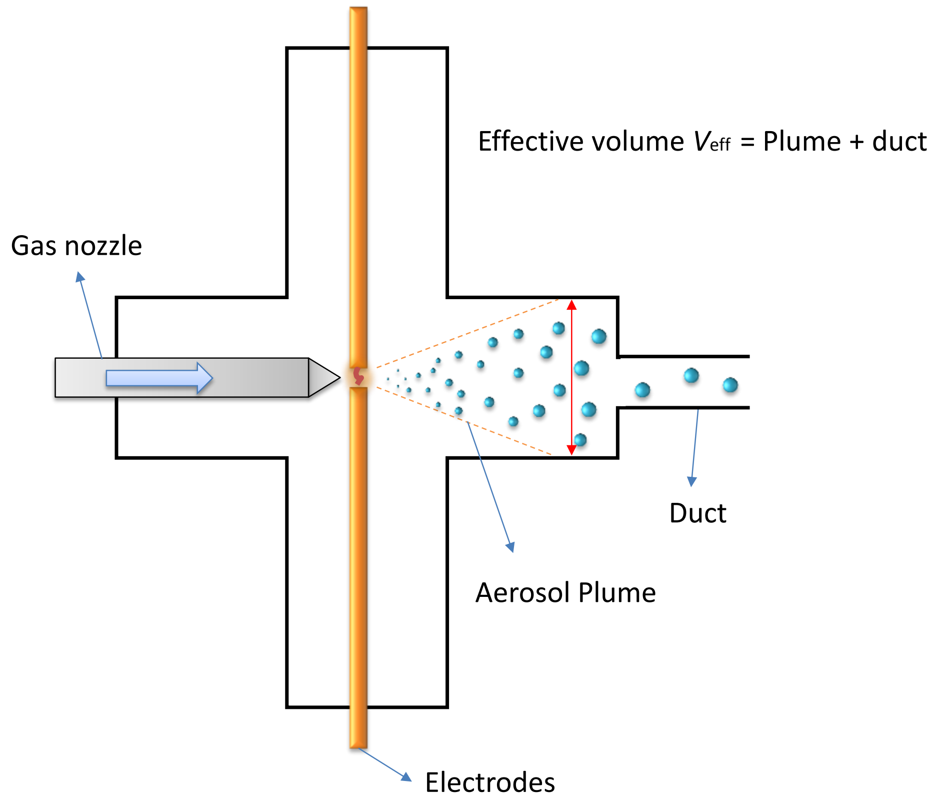

Supplementary Figure S5. Schematic illustration of particle generator indicating the effective volume V_{eff} taken up by the aerosol plume within and downstream of the generator. A gas nozzle is fixed upstream the gap between the two electrodes.

6. The critical size of singlets

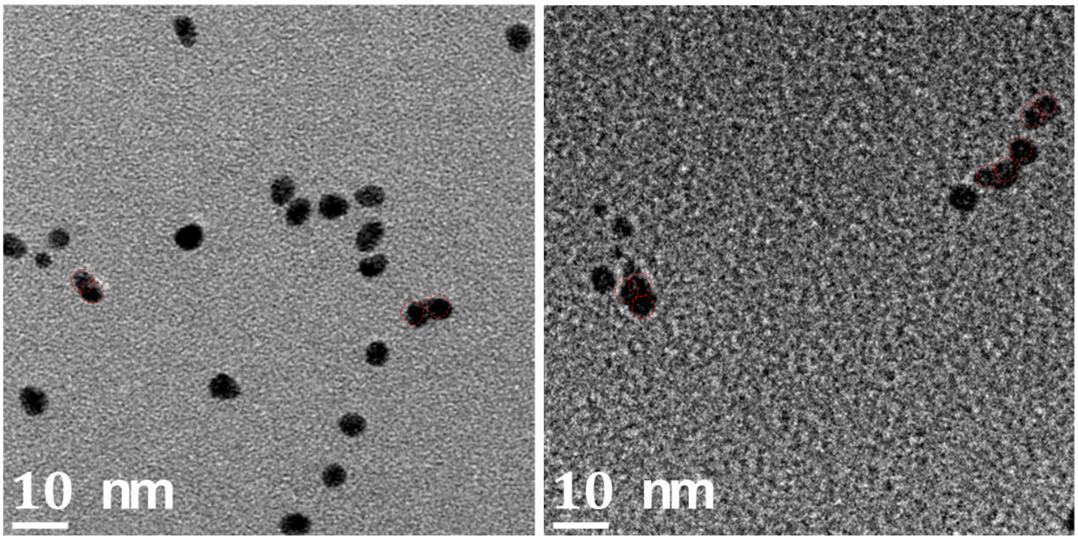

Supplementary Figure S6. TEM images of the critical size of Au singlets

Supplementary Figure S6 shows the primary particles of agglomerates having a critical size of about 5~6 nm. Comparing with the particles shown in Fig. 2, the size is the critical size for Au singlets. Subsequent collisions of particles larger than the critical size lead to the formation of agglomerates due to incomplete coalescence. The primary particles embedded in the agglomerates are the smallest round units, which have typically been considered as the minimum attainable size for ENPs.

7. Space-charge effect inside the DMA

The space charge effect is caused by the cumulative effects of the electric fields of all of the charged particles in the classification region, which can influence the DMA measurements when particle concentration is significantly high. According to Camata *et al.*, space charge increases the electric field in the particle-free region near the aerosol inlet by a factor given by[18]:

$$f_{sc} = 1 + \frac{Ne}{\varepsilon_0 V} G_F \quad (S9)$$

where N is the concentration of charged particles, e the elementary charge, ε_0 electric permittivity, V the DMA voltage, and G_F is a flow rate and geometry dependent parameter. All the relevant parameters are summarized in the Supplementary Table S3.

Supplementary Table S3. Quantities required in Supplementary equation (S9)

Parameters	Expression or value	Note
ε_0 (F m^{-1})	8.85×10^{-12}	-
G_F (m^2)	$\frac{1}{4} {r_2}^2 \left(1 - \left(\frac{r_a}{r_2}\right)^2 \left(1 - \ln\left(\frac{r_a}{r_2}\right)^2\right)\right)$	-
r_1 (m)	0.935×10^{-2}	diameter of the inner electrode of DMA
r_2 (m)	1.936×10^{-2}	diameter of the outer electrode of DMA
r_a (m)	$\sqrt{\frac{\zeta}{1+\zeta}\left({r_1}^2 + \frac{1}{\zeta}{r_2}^2\right)}$	-
ζ	$\frac{Q_a}{Q_{sh}}$	-
Q_a (slm)	1	Aerosol flow rate
Q_{sh} (slm)	14	Sheath flow rate

The space charge-effects are superimposed on the electrical field in the DMA, thereby changing the electrical mobility of the particles. Combining the work of Knutson *et al.*[19], and Camata *et al.*[18], the electrical mobility of the particle classified by a DMA when consider the space charge effect can be expressed as[19]:

$$Z_{\rm psc} = \frac{Z_{\rm p}}{f_{\rm sc}} \qquad (S10)$$

where $Z_{\rm psc}$ and $Z_{\rm p}$ represent with and without considering the space charge effect inside the DMA, respectively.

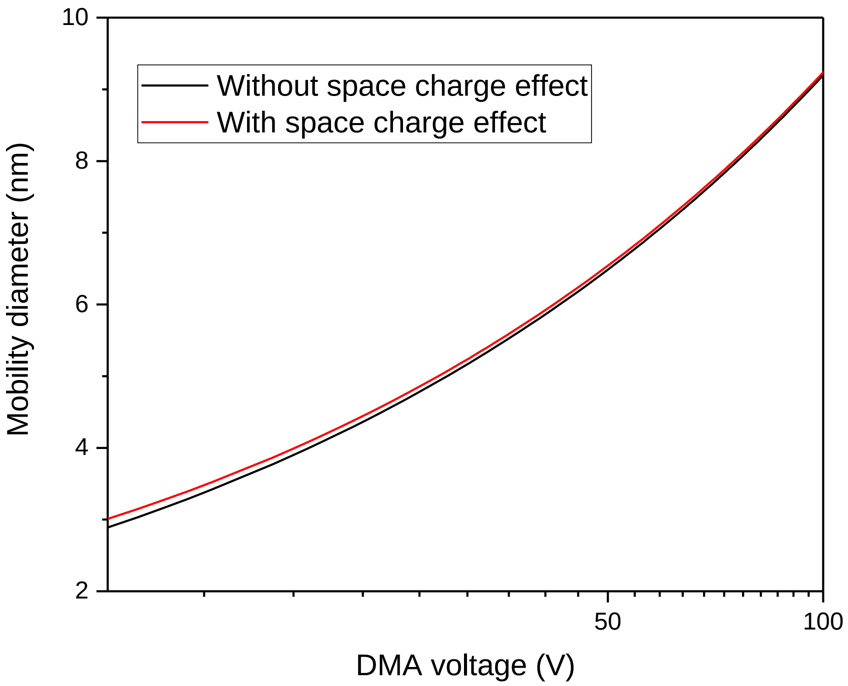

Supplementary Figure S7. Mobility diameter of particles selected by the DMA as a function voltage applied between its two electrodes with (red line) and without (black line) consideration of the space charge effect. The concentration of charged particles in these calculations is assumed to be 10^7 cm^{-3}, which is the maximum charged particle concentration derived from our measurements of the DMA and AEM.

Supplementary Figure S7 shows the space charge effect on the mobility diameters for a concentration of negatively charged particles of 10^7 cm^{-3}. The mobility diameter increases from 1 to 4 % as the particle size decreases from 6 to 3 nm (i.e., the particle size range investigated in this work). The concentration derived from the measurements using the DMA and the AEM was always below this value. It should also be pointed out that Camata *et al* have assumed a unipolarly charged aerosol[18]. Given that here we use a bipolarly charged aerosol (as produced by spark ablation), these errors are overestimated because in our case the

space charge only has an effect after the particles of the two polarities have been separated in the entrance zone of the DMA.

8. Au singlet nanoparticles deposited on substrates

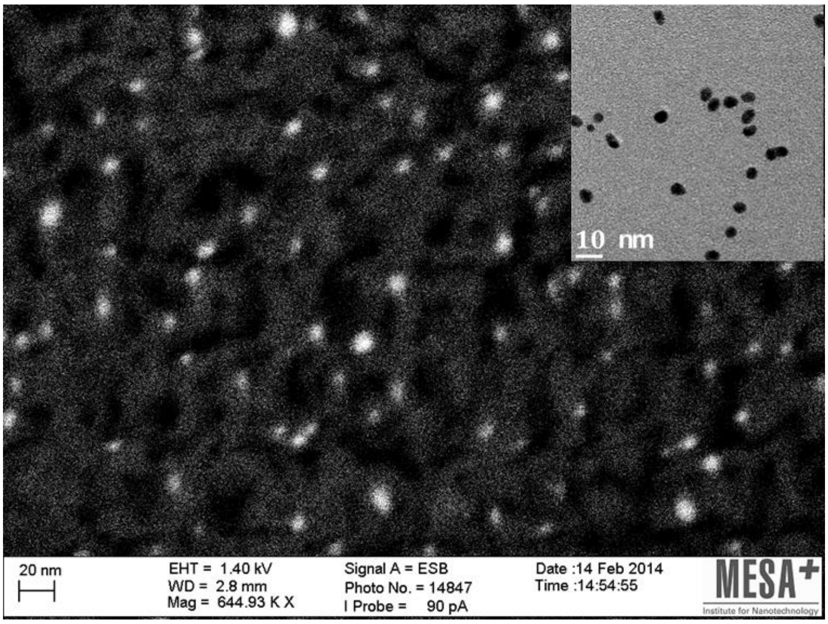

Supplementary Figure S8. SEM Micrograph of Au singlet particles deposited on a well-defined TiO_2 layer substrate. The insert is the corresponding TEM micrographs.

Supplementary Figure S8 shows SEM image of Au singlet nanoparticles produced by spark ablation distributed on a TiO_2 coated substrate. A representive TEM micrograph of particles is shown in the insert. The resulting singlet nanoparticles can be used for many applications, particularly for nanocatalysis[20]. Properly altering the process variables of spark ablation permits the ultrapure singlet Au nanoparticles to be deposited on the substrate, without using any chemical precursors or post-treatment[12].

9. Production of Ag singlet particles

Supplementary Figure S9 shows singlet Ag particles having diameters of ca. 6 nm produced by spark ablation. Due to the material properties, the production rate of Ag vapour atoms (Δmf) is ca. 1.4 times greater than that of Au (cf. Supplementary equation (S5)), which leads to particles that are approximately 1.1 times larger when using the same experimental conditions with those used for the measurements shown in Figure 2f (cf. equation (5) in the main text) under the assumption of the same value of β for both metals. The same factor is determined by comparing the singlet particles of both metals from TEM micrographs, which shows TEM analysis is in agreement with predictions using equation (5) and Supplementary

equation (S5). The comparison between model prediction and TEM analysis justifies the simple model can be expanded to other materials.

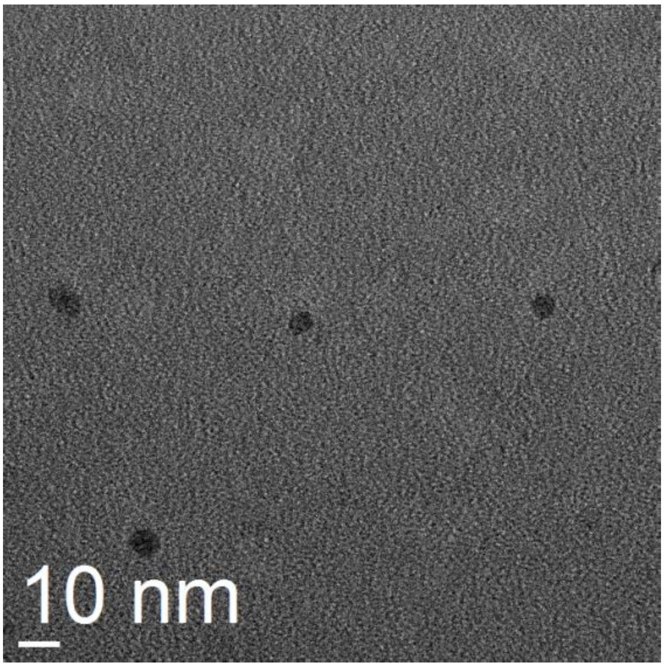

Supplementary Figure S9. TEM image of Ag singlet particles produced by spark ablation

References of Supplementary Information

1. Martinen, H. & Tholl, H. Untersuchung der temperatur und der expansion von funkenkanälen in H_2 bei variabler energiezufuhr. *Zeitschrift Naturforsch. Tl. A* **25,** 430 (1970).
2. Jenkins, N. T. & Eagar, T. W. Submicron particle chemistry: Vapor condensation analogous to liquid solidification. *JOM* **55,** 44–47 (2003).
3. Berkowitz, A. E. & Walter, J. L. Spark erosion: A method for producing rapidly quenched fine powders. *J. Mater. Res.* **2,** 277–288 (1987).
4. Gutsch, A., Mühlenweg, H. & Krämer, M. Tailor-made nanoparticles via gas-phase synthesis. *Small* **1,** 30–46 (2005).
5. Bejan, A. *Convection Heat Transfer*. (John Wiley & Sons, 2013).
6. Lai, F. S., Friedlander, S. K., Pich, J. & Hidy, G. M. The self-preserving particle size distribution for Brownian coagulation in the free-molecule regime. *J. Colloid Interface Sci.* **39,** 395–405 (1972).
7. Lee, K. W. & Chen, H. Coagulation rate of polydisperse particles. *Aerosol Sci. Technol.* **3,** 327–334 (1984).

8. De La Verpilliere, J. L., Swanson, J. J. & Boies, A. M. Unsteady bipolar diffusion charging in aerosol neutralisers: A non-dimensional approach to predict charge distribution equilibrium behaviour. *J. Aerosol Sci.* **86,** 55–68 (2015).

9. Tabrizi, N. S., Ullmann, M., Vons, V. A., Lafont, U. & Schmidt-Ott, A. Generation of nanoparticles by spark discharge. *J. Nanoparticle Res.* **11,** 315–332 (2009).

10. Hoppel, W. a. & Frick, G. M. Ion—aerosol attachment coefficients and the steady-state charge distribution on aerosols in a bipolar ion environment. *Aerosol Sci. Technol.* **5,** 1–21 (1986).

11. Llewellyn Jones, F. Electrode erosion by spark discharges. *Br. J. Appl. Phys.* **1,** 60–65 (1950).

12. Pfeiffer, T. V., Feng, J. & Schmidt-Ott, A. New developments in spark production of nanoparticles. *Adv. Powder Technol.* **25,** 56–70 (2014).

13. Seinfeld, J. H. & Pandis, S. N. *Atmospheric Chemistry and Physics: From Air Pollution to Climate Change*. (John Wiley & Sons, 2006).

14. Hinds, W. C. *Aerosol Technology: Properties, Behavior, and Measurement of Airborne Particles*. (John Wiley & Sons, 1999).

15. Ouyang, H., Gopalakrishnan, R. & Hogan, C. J. Nanoparticle collisions in the gas phase in the presence of singular contact potentials. *J. Chem. Phys.* **137,** 064316 (2012).

16. Kennedy, I. M. & Harris, S. J. Direct numerical simulation of aerosol coagulation with van der Waals forces. *J. Colloid Interface Sci.* **130,** 489–497 (1989).

17. Burtscher, H. & Schmidt-Ott, A. Experiments on small particles in gas suspension. *Surf. Sci.* **156,** 735–740 (1985).

18. Camata, P. R., Atwater, A. H. & Flagan, C. R. Space-charge effects in nanoparticle processing using the differential mobility analyzer. *J. Aerosol Sci.* **32,** 583–599 (2001).

19. Knutson, E. O. & Whitby, K. T. Aerosol classification by electric mobility: apparatus, theory, and applications. *J. Aerosol Sci.* **6,** 443–451 (1975).

20. Fraters, B. D. *TiO_2 Based Photocatalytic Gas Purification*. (University of Twente, 2015).
